# Graphene and Its Derivatives for Electrochemical Sensing

**DOI:** 10.3390/s25071993

**Published:** 2025-03-22

**Authors:** Haoliang Ren

**Affiliations:** School of Natural Science, University of Manchester, Oxford Road, Manchester M139PL, UK; renhaoliang1@gmail.com

**Keywords:** graphene and its derivatives, functional unit, electrochemical sensor

## Abstract

As a typical two-dimensional material, graphene and its derivatives exhibit many excellent properties, such as large specific surface area, electrical properties, and stability. Along with its derivatives, particularly graphene oxide (GO) and reduced graphene oxide (rGO), graphene materials have been studied in various fields due to the presence of aromatic ring, free π-π electron and reactive functional groups. This review focuses firstly on the synthesis methods of graphene and its derivatives along with their properties, followed by a discussion of the applications of their served as functional units in electrochemical sensing. Finally, this review describes the challenges, strategies, and outlooks on future developments.

## 1. Introduction

Graphene is an allotrope of carbon characterized by a single hexagonal honeycomb lattice of carbon atoms bonded through sp^2^ hybridization [1,2,3,4]. It was first isolated from graphite in 2004 by physicists Andre Geim and Konstantin Novoselov at the University of Manchester, UK [5,6]. As a nanomaterial with a distinctive two-dimensional structure, graphene exhibits remarkable strength, electrical conductivity, and thermal stability [7,8,9]. However, due to its large specific surface area, high concentrations of graphene can lead to agglomeration, resulting in uneven dispersion within composite materials and adversely affecting their performance [10,11]. Consequently, the singular form of graphene is not entirely suitable for use in sensor applications. Graphene oxide, a derivative of graphene, is relatively simple to prepare, readily available, and possesses favorable hydrophilic properties, which can mitigate some of graphene’s limitations [12,13]. Nonetheless, the abundance of oxygen-containing functional groups in graphene oxide can reduce the electron transport capacity at the modified interface, hindering electrocatalytic reactions and the development of highly sensitive sensors [14]. To enhance practical applications, it is possible to reduce and control the number of oxygen-containing groups on the surface of graphene oxide through appropriate reduction methods to produce reduced graphene oxide, which can broaden its application range to a certain extent [15]. Additionally, heteroatom doping is employed to further enhance the electrochemical sensing performance of graphene and its derivatives, improving their sensing capabilities [16,17,18]. For example, boron-doped graphene is noteworthy because boron has one less valence electron than carbon, which induces charge polarization (negative charge) within the graphene matrix, thereby favoring p-type conduction. Additionally, doping introduces extra holes into the valence band of graphene, enhancing both electrical conductivity and carrier concentration in the boron-doped graphene (BG) [19]. Nitrogen is also recognized as a high-quality doping element, primarily due to its atomic size being comparable to that of carbon atoms, which allows for the formation of strong bonds between them [20]. Incorporating nitrogen into graphene-based materials increases charge carrier density, as the p-electrons from nitrogen contribute to the π-system of graphene. Consequently, nitrogen and nitrogen-based groups play a significant role in the electrochemical investigations of nitrogen-doped materials [21].

Due to the inherent advantages of graphene and its derivatives, they are extensively utilized across various fields, including batteries [19,20,21,22], coatings [23,24,25], composites [26,27,28], electronics [29,30,31], sensors [32,33,34,35,36], biomedicine [37,38,39], and environmental protection [40,41,42]. One of the key factors contributing to their widespread application in electrochemical sensing is the ability of graphene and its derivatives to effectively integrate the benefits of individual components within composite sensing materials. The synergistic effects generated within these systems enhance the electrochemical performance of the original materials. By employing graphene in conjunction with other substances, the detection limits and sensitivity for various elements are improved while also enhancing stability and selectivity [43,44]. This underscores the broad applicability and potential of graphene and its derivatives in the realm of electrochemical sensing.

**Scheme 1:**
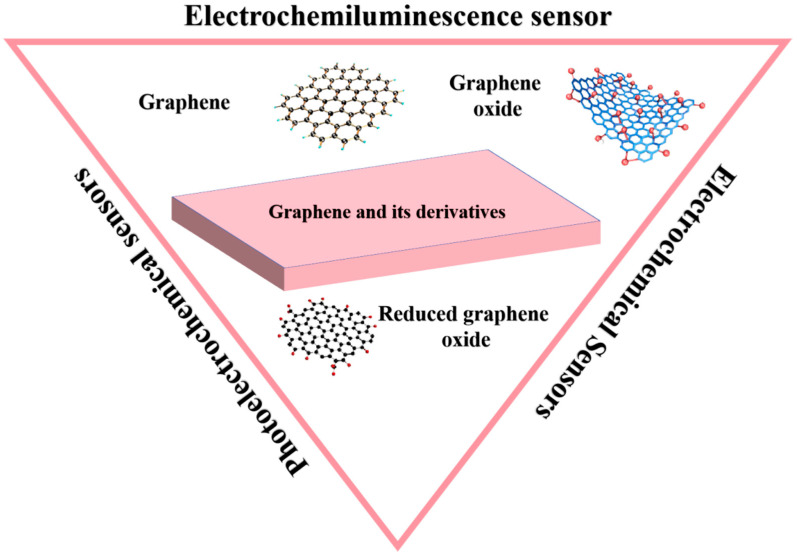
Schematic illustration of electrochemical-based sensor based on graphene and its derivatives.

Numerous reviews have addressed the sensing applications based on graphene and its derivatives [35,36,45,46,47]. For instance, Reddy et al. [46] investigated the advancements of graphene composites for electrochemical sensors from both structural and functional perspectives, while Coros et al. [47] examined the progress of graphene-based materials for enhanced biosensors with a focus on improved analytical performance. In this discussion, we will explore the research advancements related to the application of graphene and its derivatives in electrochemical sensing, photoelectrochemical sensing, and electrochemiluminescence sensing, emphasizing their role as functional units (Scheme 1).

## 2. Graphene and Its Derivatives Preparation

The preparation and modification of graphene and its derivatives significantly influence their properties and applications. Efforts have been directed towards various methods of synthesis and property alteration to optimize their compatibility with different sensor substrates. The ultimate goal is to maximize their efficiency, which consequently leads to enhanced performance of the sensor devices.

### 2.1. Graphene

Since the advent of graphene, researchers have been actively investigating methods for the rational preparation of graphene and its derivatives. Currently, there are two primary approaches to synthesizing graphene: (1) the separation of intact bulk graphite into graphene or its derivative products [48,49] and (2) the utilization of a carbon source to facilitate the synthesis of graphene and its derivatives [50,51]. Graphene produced through contemporary synthesis techniques exhibits increasing purity compared to graphene derived from initial isolation methods. The principal current synthesis methods are summarized in Table 1.

**Micromechanical Exfoliation**. In 2004, Geim et al. pioneered the micromechanical exfoliation technique, successfully isolating and characterizing a monolayer of graphene from highly oriented pyrolytic graphite for the first time [5]. This groundbreaking work elucidated the quasi-two-dimensional nature of graphene, revealing the underlying principles governing its unique two-dimensional crystal structure. Micromechanical exfoliation represents the earliest approach for graphene fabrication, primarily involving the application of mechanical forces to the surface of freshly cleaved graphite crystals to generate graphene flakes. These flakes are subsequently transferred onto a substrate, facilitating the acquisition of graphene. Novoselov et al. [5] further advanced this technique in 2004 by employing transparent adhesive tape to repeatedly exfoliate highly oriented pyrolytic graphite, thereby bridging the gap in the exploration of two-dimensional carbon materials. Their findings affirmed the feasibility of isolating graphene crystals as discrete entities [52,53]. Despite the accessibility and convenience of the micromechanical exfoliation method, it is not without significant drawbacks. While the technique yields high-quality graphene, it is characterized by a low production rate and high associated costs, rendering it unsuitable for industrial-scale mass production. Consequently, micromechanical exfoliation is best suited for laboratory-scale synthesis of graphene for research applications.

**Epitaxial Growth**. This preparation technique employs silicon carbide (SiC) and densely arranged metals as substrates to cultivate carbon-rich surfaces [54,55,56]. This is achieved through a process in which SiC is heated in an ultra-high vacuum (UHV) environment at temperatures ranging from 1273 K to 1773 K [55,56]. This method also facilitates the generation of graphene at the wafer level. The primary advantages of SiC epitaxial graphene include the elimination of the need for transfer during device processing and the ability to produce graphene sheets that can be as large as the substrate itself. However, the preparation conditions for the SiC epitaxial growth method are more stringent than those of other methods, presenting challenges in completely separating the graphene from the substrate and minimizing defects. Zebardastan et al. [55] utilized face-to-face annealing of SiC in an ultrahigh vacuum to exert control over the epitaxial graphene process, successfully obtaining a single graphene layer characterized by a smooth surface, high purity, and virtually defect-free quality.

**Chemical Vapor Deposition (CVD)**. CVD is a method used to deposit graphene onto a transition metal substrate [57]. In this process, methane or other carbon-containing gases are introduced into a furnace at elevated temperatures ranging from 750 °C to 1080 °C, with hydrogen serving as a reducing agent and argon acting as a protective gas. The high-temperature environment promotes the decomposition of methane, leading to the formation of graphene on the substrate surface, where the transition metal substrate also functions as a catalyst during the reaction [58,59]. CVD is a well-established technique for graphene production, recognized for its stability and the ability to produce large, uniform films. Although graphene generated through CVD can be transferred to other insulating substrates, effective transfer methods have not yet been fully developed, making this process more complex and resulting in increased production costs. Despite these challenges, CVD remains a widely adopted approach for the mass production of graphene. Sun et al. [60] successfully demonstrated the mass production of A3-sized (0.42 × 0.3 m^2^) single-crystal graphene films on Cu(111) foils using a custom-designed medium-scale CVD system. The resulting product exhibited ultra-high carrier mobility.

Overall, the average cost associated with these graphene production methods remains high. The future production of graphene continues to encounter numerous challenges. These cost-related issues may lead to problems such as resource wastage in large-scale graphene preparation. To advance the exploration and study of graphene production, researchers must further investigate the precise control of production processes associated with these methods. Emphasis should be placed on developing newer and more rational procedural steps and improving raw material handling during the synthesis of graphene.

### 2.2. Graphene Oxide

Graphene oxide (GO) is a derivative of graphene characterized by the incorporation of numerous oxygen-containing functional groups on its surface and edges. The presence of these functional groups not only enhances its reactivity compared to graphene but also improves its stability in water and polar solvents [61,62]. Currently, there are several well-established chemical oxidation methods for producing graphene oxide, including Brodie’s method, Staudenmaier’s method, and Hummers’ method. All the information summarized in Table 2.

**Brodie’s Method**. In 1859, Brodie’s method yielded the first graphene oxide through the reaction of potassium chlorate, graphite powder (commonly sourced from natural graphite flakes), and concentrated nitric acid [63]. This approach is relatively straightforward and employs readily available raw materials; however, it necessitates the use of harsh reaction conditions.

**Staudenmaier’s Method**. In 1898, Staudenmaier advanced Brodie’s original approach by integrating concentrated sulfuric acid with the nitric acid initially utilized by Brodie [64,65]. The enhanced oxidative capabilities of the mixed acids significantly accelerated the reaction, resulting in improved yields of graphene oxide. Staudenmaier’s modifications streamlined the preparation process and provided greater control over the extent of the reaction. However, this method is marked by extended reaction times and requires elevated temperatures.

**Hummers’ Method**. In 1958, Hummers and Offeman introduced a method for synthesizing graphene oxide that utilizes potassium permanganate and sodium nitrate in a sulfuric acid medium [66]. This technique not only retains the operational simplicity of both Brodie’s and Staudenmaier’s methods but also improves the yield and quality of graphene oxide while ensuring a high safety profile and effective oxidation capabilities. Although Hummers’ method is more complex, it has emerged as the most widely adopted approach in contemporary applications.

Despite their advantages, these techniques share a significant drawback: they produce several toxic gases, including nitrogen dioxide and nitrogen tetroxide, which pose environmental hazards. In some cases, chlorine dioxide is also generated as a by-product, which is both explosive and harmful to experimental or industrial processes. Over the years, improvements to the Hummers’ method have focused on identifying superior raw materials to replace sodium nitrate or potassium permanganate. For instance, Chen et al. [67] demonstrated that nearly identical graphene oxide could be synthesized without sodium nitrate, while Pen et al. [68] employed the stronger oxidizing agent K_2_FeO_4_ as a substitute for KMnO_4_. This latter approach not only reduces the introduction of heavy metals and the emission of toxic gases during synthesis but also facilitates the recycling of sulfuric acid. Consequently, this method is regarded as green, safe, efficient, and cost-effective, yielding graphene oxide powder that exhibits excellent solubility in both water and polar organic solvents.

In addition to conventional methods, researchers have also investigated the use of organic compounds as oxidizing agents and electrochemical techniques for the synthesis of graphene oxide. For instance, Shen et al. [69] utilized benzoyl peroxide as an oxidizing agent, capitalizing on its low melting point and employing it as a solvent during the oxidation process to generate graphene oxide. However, the application of benzoyl peroxide raises safety concerns due to its potential for explosive decomposition when subjected to heat in a sealed container.

At this juncture, neither these alternative methods nor the original Hummers’ method can achieve a complete conversion from graphene to graphene oxide [66]. Although a method for full conversion remains elusive, the optimization of reaction conditions and the exploration of various synthesis techniques can enhance the conversion rate and purity, ultimately yielding higher-quality graphene oxide.

### 2.3. Reduced Graphene Oxide

Reduced graphene oxide (rGO) is synthesized through the reduction of graphene oxide, a process that involves the removal of oxide functional groups [15]. While rGO retains certain properties characteristic of graphene, such as electrical conductivity, the reduction process often results in the retention of impurity atoms, including residual oxygen, as well as the introduction of structural defects. Consequently, the quality of rGO does not reach that of pristine graphene, although it remains suitable for specific applications. In recent years, the preparation of reduced graphene oxide has attracted increasing interest from the scientific community, particularly with respect to the selection of effective reduction methods for rGO synthesis. Table 3 summarizes various reduction methods for graphene oxide.

**Thermal Annealing Reduction**. The thermal reduction method involves the rapid heating of graphite oxide in a vacuum or an inert/reducing gas atmosphere, effectively facilitating the deoxygenation process and yielding graphene [71]. This technique addresses both internal and external oxygen-containing functional groups. The swift temperature rise induces the decomposition of these functional groups into gaseous byproducts, generating pressure between the stacked layers, which in turn promotes delamination [73,74,75]. While this method minimizes contamination, the release of carbon dioxide during thermal reduction can lead to structural degradation of the graphene layers. Additionally, the high temperatures required for this process entail significant energy consumption and present challenges, particularly for certain low-melting-point substrates that may not endure the conditions. Saleem et al. [85] employed this method to reduce a modified version of Hummers-prepared graphite oxide at 500 °C, demonstrating a substantial removal of oxide groups; however, some hydroxyl and cyano groups remained attached to the rGO layers due to incomplete reactions.

**Photoreduction**. Photoreduction requires an optical medium, such as lasers or plasma. One variant of this technique is photothermal reduction, which utilizes high-energy light to induce rapid localized heating of graphene oxide (GO), thereby facilitating the decomposition of oxygen-containing functional groups. In contrast, plasma reduction generates charged particles that bombard the GO through low-energy inelastic collisions, effectively cleaving the oxygen-containing groups from the GO surface [76]. Photoreduction offers significant advantages in scalability and cost-effectiveness, particularly allowing for precise control over the localized reduction of GO within a short time frame. However, this method does not address the issue of defects within the GO substrate, and the necessity for advanced experimental equipment, such as lasers or plasma sources, limits its broader applicability. De Lima et al. [86] demonstrated enhanced reduction outcomes by employing a combination of ultraviolet (264 nm) and infrared (1066 nm) irradiation, achieving an oxygen content of less than 1% post-reduction.

**Microwave Reduction**. Microwave reduction employs microwave radiation to heat materials, resulting in the gradual loss of oxygen-containing functional groups as the temperature rises, while preserving the stability of the rGO sheets [78]. This technique has emerged as a rapid reduction method. Unlike traditional thermal convection heating, microwaves directly heat materials through dielectric losses, thereby enhancing heating efficiency. Microwave reduction can be executed independently or combined with chemical and thermal reduction methods to accelerate the reduction process. This approach facilitates shorter reduction times and offers improved control over the reduction temperature. However, further advancements are required for large-scale applications, as the intricacies of microwave heating necessitate a more comprehensive understanding of the material’s dielectric properties as a function of temperature, along with enhancements in reduction quality through precise process control [79]. For instance, Voiry et al. [80] successfully generated large rGO sheets with transverse dimensions reaching several tens of micrometers by irradiating graphene oxide (GO) with short microwave pulses of 1–2 s, following an annealing process at 300 °C for one hour in an argon atmosphere.

**Chemical Reduction**. Chemical reduction is less demanding in terms of equipment and environmental conditions, making it a viable method for large-scale production due to its cost-effectiveness and simplicity [83]. Chemical reduction involves the reaction of a chemical reagent with graphene oxide (GO), typically taking place at room temperature or with mild heating. The selection of reductants is critical for controlling the quality of the reduction process. Hydrazine has been employed for the reduction of graphite oxide since before the emergence of graphene and is now recognized as an effective reagent for reducing graphene oxide [82,83,87]. This reduction process, which can be carried out using hydrazine and its derivatives (e.g., hydrazine hydrate and dimethylhydrazine), entails the addition of liquid reagents to aqueous dispersions of GO. This results in increased hydrophobicity and the formation of aggregated graphene-based nanosheets, which ultimately dry into a black powdery conductor [84,88]. However, careful attention must be given to the selection of materials, as reducing agents such as hydrazine are toxic and may introduce doping into the final product, as well as generate waste that can elevate industrial production costs [81]. Recently, sodium borohydride (NaBH4) has emerged as a more efficient reducing agent for GO compared to hydrazine [89]. Furthermore, Cao et al. [90] have proposed improving the moderate efficiency of NaBH4 in reducing epoxy and carboxylic acids by utilizing concentrated sulfuric acid following the reduction with NaBH4 and implementing a dehydration process at temperatures below 180 °C.

Overall, the structure of reduced graphene oxide is intricately linked to the reduction strategies employed, and promising results have emerged from ongoing research. In the near future, researchers will not only develop a diverse array of reduction methods to enhance productivity, but they will also be able to utilize other low-cost and environmentally friendly reducing agents, which will greatly facilitate the process.

### 2.4. Green Synthesis of Graphene and Its Derivatives

The synthesis of graphene and its derivatives inevitably raises environmental concerns. During the synthesis process, various issues arise, including pollution from chemical reagents, waste, and by-products, as well as ecotoxicity [91,92]. As previously mentioned, the choice of reducing agent is a critical factor in the production of rGO. In recent years, researchers have explored numerous green reducing agents as alternatives to traditional ones, aiming for more sustainable production methods. For instance, Lam et al. [93] utilized banana peel extracts—rich in phenolic compounds, alkaloids, flavonoids, and vitamin C, all of which have demonstrated antioxidant properties [94,95]—as a green reducing agent, aided by ultrasound assistance. While their results confirm that banana peel extract is a viable reducing agent, it still falls short of the effectiveness demonstrated by hydrazine hydrate, indicating a need for further research to enhance reduction efficiency.

Moreover, considering the green production of graphene and its derivatives solely from the perspective of raw materials is not an entirely effective or environmentally friendly approach. Wang et al. [96] reported a novel method for producing RGO, which involves mixing biomass such as wood, straw, starch, bamboo, or grass with concentrated sulfuric acid to generate rGO. This approach not only addresses the challenge of managing large quantities of waste biomass but also avoids the production of hazardous waste mixtures and the use of toxic or flammable reducing agents.

## 3. Graphene and Its Derivatives for Electrochemical Sensing

Currently, graphene and its derivatives represent highly promising application areas within the sensor field due to their excellent electrical conductivity and chemical properties [97,98]. These characteristics render graphene suitable not only as a conductive material but also as a substrate for conductive materials. By incorporating graphene, certain sensors can attain superior performance and sensing efficiency, thanks to its higher and more uniform chemically active sites compared to other similar electrode materials. However, the conductivity and electrocatalytic activity of graphene and its derivatives can be compromised by factors such as agglomeration [10,11,98]. Over the years, there has been a trend of combining graphene and its derivatives with metals, metal oxides, polymers, and other materials to create composite nanomaterials that enhance their properties [99,100,101]. Recent advancements in electrochemical sensing utilizing graphene and its derivatives, as well as nanomaterial/graphene composites as functional materials, are discussed below.

### 3.1. Electrochemical Sensor

An electrochemical sensor is a device that converts the presence or concentration of a chemical substance into an electrical signal [102]. These sensors are extensively employed in biochemical monitoring, environmental analysis, and medical diagnostics. Within these sensing frameworks, graphene and its derived composites can be integrated into effective functionalized sensor modules, thereby enhancing the detection and sensing performance.

**Figure 1:**
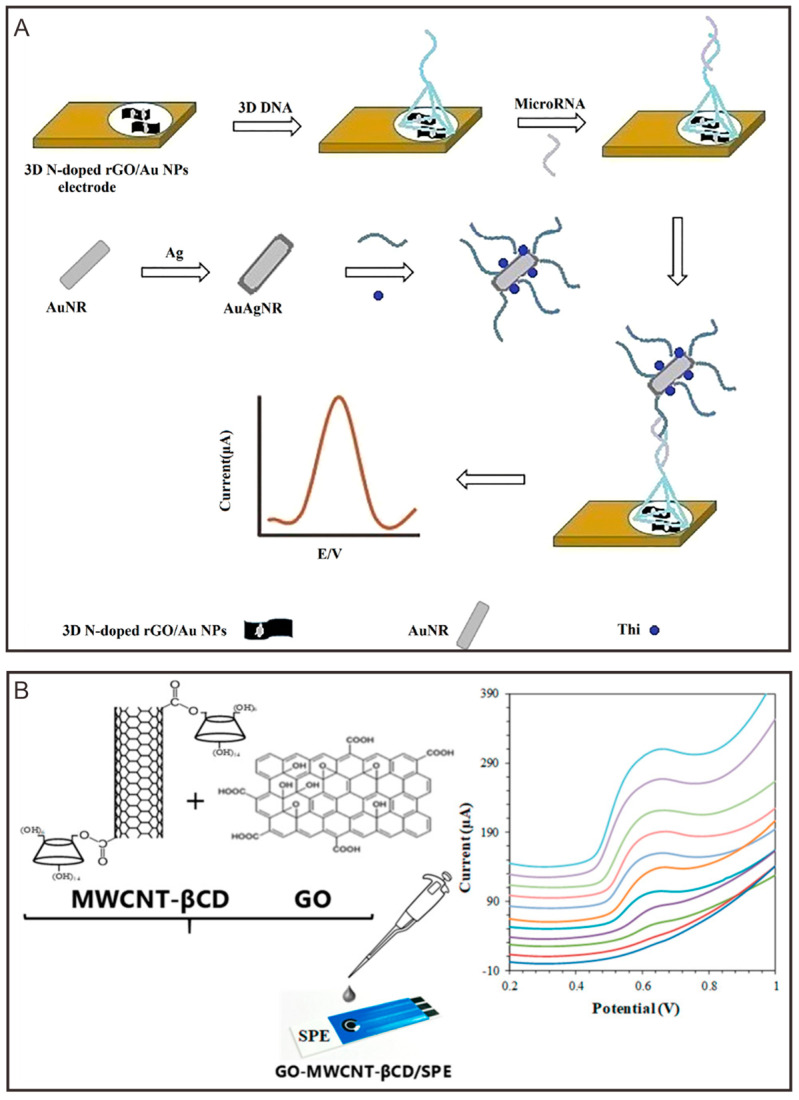
Graphene and its derivatives participate in the construction of electrochemical sensors. (**A**) Schematic diagram of electrochemical detection of miRNA-155 [103]. Copyright 2018, Elsevier B.V. (**B**) Carbon electrode modified by MWCNT-βCD composite and corresponding electrochemical signals [104]. Copyright 2020, American Chemical Society.

Tian et al. [103] reported the development of an electrochemical biosensor utilizing three-dimensional nitrogen-doped reduced graphene oxide/gold nanoparticle composite electrodes for the detection of miRNA-155 (Figure 1A). In this configuration, reduced graphene oxide (rGO) serves as a high-surface-area substrate for immobilizing the probe, facilitating target capture. The substantial contact surface area of rGO in the overall structure not only enhances the immobilization of the DNA probe but also promotes efficient electron transfer during the detection process, leading to high-sensitivity performance. The application of graphene-like materials represents a promising new direction for electrochemical environment sensing. Alam et al. [104] successfully developed multi-walled carbon nanotube (MWCNT)-β-cyclodextrin (β-CD) composites based on graphene oxide (GO) and covalent functionalization (Figure 1B), which were subsequently employed to modify the carbon electrodes of the sensor. In the composites utilized for the sensor, GO contributed not only excellent electrical conductivity but also increased the contact surface area. The incorporation of GO markedly improved the electrical conductivity and electron transfer capabilities of the MWCNT-βCD composite matrix. Furthermore, the presence of GO transformed the original monolayer adsorption of bisphenol A (BPA) by pure MWCNT species into multilayer surface adsorption, enabling greater BPA adsorption and thus enhancing detection sensitivity. The sensor demonstrated high stability, reproducibility, and selectivity for BPA detection. Notably, the electrochemical sensor maintained robust sensing performance in the presence of various interfering substances, achieving recovery rates of 96% to 105%, indicating promising potential for water quality testing applications.

Based on the modular application of the composites of graphene and its derivatives in electrochemical sensors, it is anticipated that these materials will leverage their outstanding conductivity and electron transfer capabilities when integrated into sensor electrodes. Additionally, the high adsorption properties of graphene and its derivatives can be utilized to enhance the binding of sensor receptors, thereby improving sensor efficiency. Table 4 shows examples of graphene and its derivatives used for electrochemical sensing.

### 3.2. Photoelectrochemical Sensor

The photoelectrochemical process involves the conversion of light energy into electrical energy through charge transfer, which is initiated by the excitation of electrons due to the absorption of photons by molecules, ions, or semiconductor materials [108]. The photoelectrochemical sensing method is an optical–electrochemical analytical technique that relies on the light-induced electron transfer process occurring at the electrode/solution interface [109]. This method inherits key characteristics of electrochemical detection, such as high integration and low cost. Notably, the excitation and response signals in photoelectrochemical sensing differ, resulting in a high signal-to-noise ratio and heightened sensitivity, comparable to that of electrochemiluminescence methods [110].

**Figure 2:**
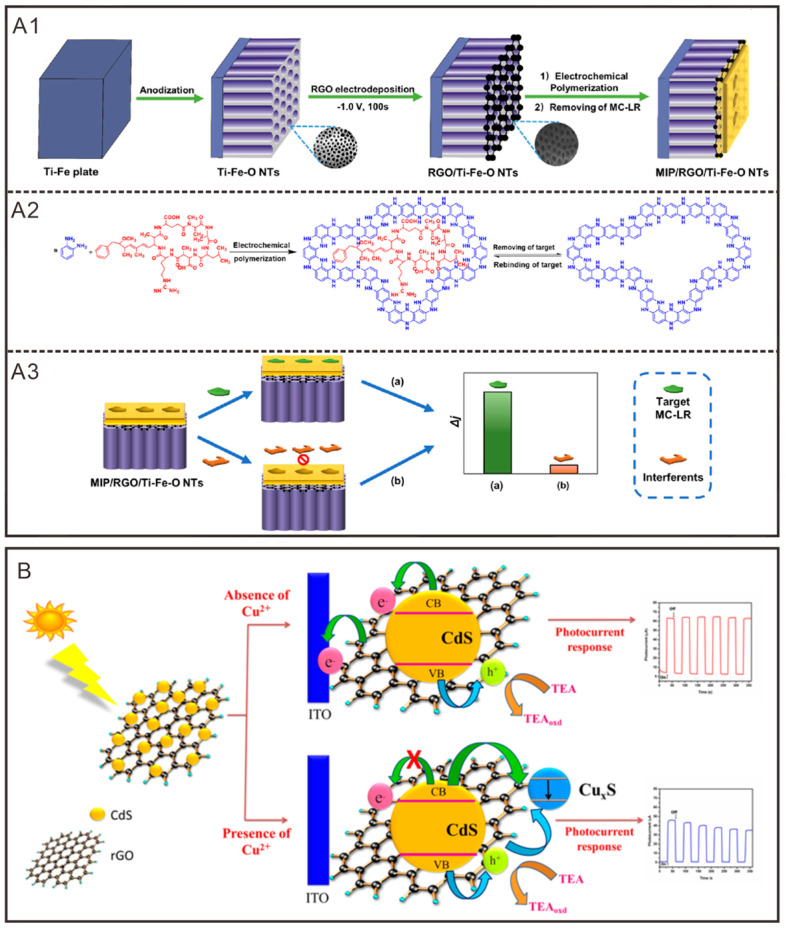
Graphene and its derivatives participate in the construction of photoelectrochemical sensors. (**A1**–**A3**) Schematic diagram of PEC sensor construction based on MIP/rGO/Ti-Fe-O NTs and target recognition mechanism of MIP [111]. Copyright 2021, American Chemical Society. (**B**) Mechanism of Cu^2+^ ion induction based on ITO/CdS-rGO nanocomposites [112]. Copyright 2016, Public Library of Science.

In their study, Song et al. [111] described a photoelectrochemical sensor platform that utilizes graphene functionalization (Figure 2A1–A3). This platform is based on molecularly imprinted polymer/rGO/Ti-Fe (MIP/rGO/Ti-Fe) nanotube (NT) electrodes, with rGO serving as a bridge among the sensors. Photogenerated electrons migrate from rGO to Ti-Fe NTs, while holes move in the opposite direction through the same carrier. The exceptional conductivity and π-π conjugated structure of rGO significantly enhance the carrier migration rate, resulting in improved sensor conductivity. Additionally, the study revealed that potential interactions between the hydroxyl and carboxyl groups on the surface of rGO and the Ti-O framework of TiO_2_ may enhance the interfacial stability between graphene oxide and Ti-Fe NTs. Consequently, the sensor demonstrated good conductivity and light responsiveness, as well as high sensitivity and selectivity towards pollutants, offering a promising photoelectrochemical sensing platform for the analysis of various contaminants.

Ibrahim et al. [112] examined an optical chemical sensing platform for copper ions based on rGO-modified photoelectrodes (Figure 2B). They deposited CdS nanoparticles, a photoactive material, onto the surface of rGO. The incorporation of rGO not only mitigated the rapid photogenerated electron–hole recombination of CdS, thus enhancing its photocurrent output, but also leveraged its intrinsic advantages in photoelectrochemical sensing, including high surface functionality, elevated electrochemical activity, and superior charge transfer efficiency. Furthermore, this approach addressed the issue of photocorrosion associated with CdS nanoparticles. The results demonstrated an excellent photocurrent response potential, yielding a significant signal upon targeting Cu^2+^ ions. This sensor exhibits considerable promise for practical applications.

Likewise, the application of the composite of graphene and its derivatives in PEC sensors is increasingly emphasizing the conductive electrode. The advantages of graphene and its derivatives are particularly evident in their exceptional electrical conductivity and conjugate structure, which enhance carrier transfer efficiency during the sensing process, thereby improving the overall efficiency of the sensor. Table 5 shows examples of graphene and its derivatives used for electrochemical sensing.

### 3.3. Electrochemiluminescence Sensor

The response mechanism of electrochemiluminescence sensors is rooted in the electrochemiluminescence (ECL) reaction. Electrochemiluminescence refers to the luminescent phenomenon that occurs due to electron transfer between charged species, driven by electrochemical excitation and high-energy electron transfer [116]. More specifically, electrochemical luminescence involves the activity of a chemiluminescent substance at a predetermined potential, where the presence of redox-active species in solution facilitates the transition from an unstable excited state back to the ground state.

**Figure 3:**
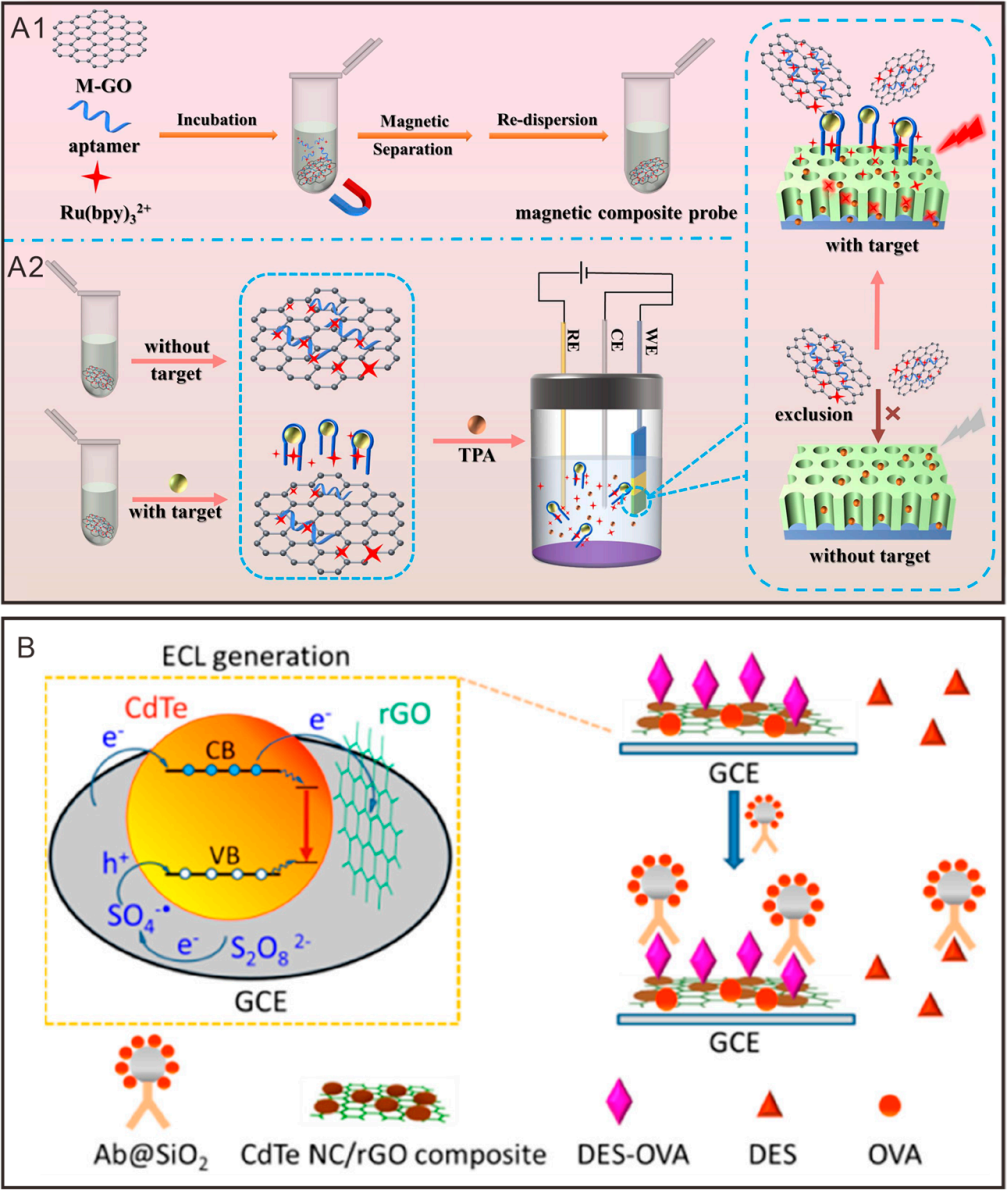
Graphene and its derivatives participate in the construction of electrochemiluminescence sensors. (**A1**,**A2**) Preparation of the magnetic composite probe and schematic diagram of the homogeneous ECL aptamer sensor platform based on the magnetic composite probe and VMSF/ITO electrode for detecting the target [117]. Copyright 2024, Elsevier B.V. (**B**) Schematic diagram of electrochemical Luminescence (ECL) of CdTe nanocrystals (NC)/rGO composite films [118]. Copyright 2020 American Chemical Society.

Electrochemical luminescence sensors have evolved from simple detection devices to sophisticated stand-alone chemiluminescent systems. Zhou et al. [117] established a homogeneous ECL aptamer sensor platform that operates without fixation or labeling (Figure 3A1,A2). This platform comprises a magnetic composite probe and a vertically ordered mesoporous silica film (VMSF)-modified electrode, where the magnetic composite probe utilizes M-GO as the adsorbent material. M-GO exhibits strong magnetic properties and a high density of oxygen-containing functional groups, making it versatile for various applications and enabling the construction of high-sensitivity sensors. In this configuration, the adsorption capacity of M-GO mitigates background signals, while the significant enhancement of the ECL signal by VMSF/ITO ultimately results in high sensitivity and low detection limits for alpha-fetoprotein (AFP) and prostate-specific antigen (PSA). This sensor offers an efficient and highly sensitive approach for detecting trace biomarkers in clinical bioanalysis.

Similarly, Shan et al. [118] developed an electrogenerated chemiluminescent composite film of CdTe nanocrystals/rGO for the detection of ethylestradiol (Figure 3B). In this sensor, rGO serves as a carrier, effectively mixed with the CdTe nanocrystals to form the composite film. The ECL emission of the composite film is significantly enhanced due to rGO’s facilitation of electron transfer during the sensing process, aided by the adsorption of dissolved oxygen (acting as a co-reactant). Furthermore, rGO induces modifications in the energy band structure of the CdTe nanocrystals, particularly by filling trap states, which contributes to the overall enhancement of the luminescent signal. These findings may inspire the development of novel ECL-based biosensors.

In the realm of ECL sensors, graphene and its derivatives are extensively employed for the adsorption of sensing receptors and the enhancement of sensing signals (Table 6). Specifically, the incorporation of graphene and its derivative materials facilitates the preparation of a diverse array of functional probes for electrochemiluminescence (ECL), enabling the detection of highly sensitive target analytes. Moreover, these materials also enable sensitive signal transmission in scenarios requiring precise detection, underscoring the significant contributions of graphene materials to the future advancement of ECL technology.

## 4. Summary and Challenges

Graphene and its derivatives, integral to numerous modern applications worldwide, exhibit remarkable strength, electrical conductivity, thermal stability, and a high specific surface area. This paper reviews the various preparation methods for graphene and its derivatives, as well as their applications in electrochemical sensors, photoelectrochemical sensors, and electrochemiluminescence sensors. Notably, graphene and its derivatives can significantly enhance sensor performance, whether used independently or incorporated into composites to form functional units within the sensors. Specifically, they serve as effective adsorption substrates, sensing probes, and conduction electrodes, thereby improving the overall performance of the sensors.

Understanding how graphene composites can enhance the efficiency of sensors is now crucial for the advancement of new, sophisticated sensor products. Graphene and its derivatives hold significant promise for sensor applications; however, many functionalized sensors incorporating graphene and its derivatives remain in the experimental phase and are not yet commercially available.

**Synthesis:** One of the primary challenges is the large-scale production of high-quality graphene, necessitating the development of more efficient synthetic strategies. A key obstacle in the synthesis process is the ability to effectively manipulate the properties of graphene through various methods. Although machine learning can guide the large-scale synthesis of graphene and its derivatives, there are several issues: (1) there are limited experimental data on the synthesis of high-quality graphene, especially regarding the process parameters and performance correlation data for specific derivatives (such as graphene oxide and fluorinated graphene); (2) the synthesis of graphene involves multi-scale correlations from atomic-level reactions (such as precursor decomposition in CVD growth) to macroscopic process parameters (temperature, pressure, gas flow rate); (3) black-box models (like deep neural networks) struggle to provide physical explanations of the synthesis mechanisms, hindering experimental optimization; (4) the synthesis paths predicted by machine learning require time-consuming and costly experimental validation (such as the operation of high-vacuum CVD equipment).

**Application:** There is a pressing need to explore cost-effective and environmentally friendly production techniques for graphene and its derivatives. When considering the application of graphene-functionalized modules in a variety of sensors, it is essential not only to recognize their immense potential in the sensor domain but also to leverage their properties effectively in modular applications. This approach is critical for the development of sensors that are more efficient, stable, and durable.

**Commercialization:** In the commercialization process of graphene sensor products, we also need to solve the industry standardization issues of related products but also to overcome possible regulatory barriers to products.

## Figures and Tables

**Table 1:** The preparation methods of graphene.

Methods	Conditions	Advantages	Disadvantages	References
Mechanical Exfoliation	-Mechanical peeling	-Simple method -High quality	-Repeated stripping -Low yield -High cost	[5,52,53]
Epitaxial Growth	-Specific atmosphere -Heat treatment	-Large-scale preparation	-Harsh conditions -difficult separate	[54,55,56]
CVD	-Metal substrate -Carbonaceous gas -High temperature	-Catalyst -Transferable substrate	-Inefficient transfer -High cost	[57,58,59]

**Table 2:** The preparation methods of graphene oxide.

Methods	Conditions	Advantages	Disadvantages	References
Brodie	-Reducing agent -Carbon source -Strong acid -Slow heat	-Simple method -Common ingredients	-Harsh reaction conditions -Disunity of quality -Uncontrol	[63]
Staudenmaier	-Reducing agent -Strong mixed acid -Carbon source	- Oxidizing raw material more -Controllable reaction	-High temperature -Time consuming	[64,65]
Hummer	-Strong reducing agent -Strong mixed acid -Carbon source -Heating	-Large output -High quality	-Difficult operation -Harmful to the environment	[66]

**Table 3:** The preparation methods of reduced graphene oxide.

Methods		Conditions	Advantages	Disadvantages	References
Thermal Annealing Reduction		-Vacuum or inert environment -Rapid heating	-Uniform reaction -Promotes stripping -Less impurities	-Product loss -High energy consumption	[70,71,72,73,74,75]
Photoreduction	Photothermal	-High-energy light	-High-quality workmanship -Time-controlled -Low-cost -Locally efficient	-Product defects -High equipment requirements	[76,77]
	Plasma	-Charged particles colliding			
Microwave reduction		-Microwave radiation	-Controlled time -High purity -Short time -Practicality	-Complex heating processes	[78,79,80]
Chemical reduction		-Room temperature or moderate heat -Reducing agent	-Fast -Efficient -Low reduction requirements -Low cost	-Toxic by-products -Environmental hazards	[81,82,83,84]

**Table 4:** Graphene and its derivatives for electrochemical sensing.

Materials	Linear Range (M)	Detection Limit (M)	References
3D N-doped rGO/AuNPs	1 × 10^−11^–1 × 10^−4^	1 × 10^−12^	[103]
MWCNT-βCD/GO	5 × 10^−8^–5 × 10^−6^ /5 × 10^−6^–3 × 10^−4^	6 × 10^−9^	[104]
PA6/CNW:rGO	2.5 × 10^−6^–2 × 10^−4^	5.2 × 10^−9^	[105]
ZrP/GO	8× 10^−9^–2.6 × 10^−5^	1 × 10^−9^	[106]
NH_2_–UiO-66/RGO	2× 10^−8^–1 × 10^−6^	6.67 × 10^−9^	[107]

PA6: polyamide 6, CNW: cellulose nanowhiskers, ZrP: zirconium phosphate.

**Table 5:** Graphene and its derivatives for photoelectrochemical sensing.

Materials	Linear Range (M)	Detection Limit (M)	References
RGO/Ti–Fe–O NTs	5 × 10^−11^–5 × 10^−8^	1 × 10^−11^	[111]
CdS-rGO	5 × 10^−7^–1.2 × 10^−4^	1.6 × 10^−8^	[112]
SnS_2_/AgBiS_2_/GO	1 × 10^−8^–5 × 10^−5^	4 × 10^−9^	[113]
CoFe_2_O_4_/CuTAPc-GO	2.5 × 10^−7^–3.2 × 10^−4^	1.6 × 10^−8^	[114]
CMP-rGO	5 × 10^−9^–4 × 10^−5^	2.7 × 10^−9^	[115]

CuTAPc: copper tetraaminophthalocyanine, CMP: conjugated microporous polymer.

**Table 6:** Graphene and its derivatives for ECL sensing.

Materials	Linear Range (M)	Detection Limit (M)	References
M-GO/VMSF	0.01–100 pg/mL	9.6 pg/mL	[117]
GO/ZnS	1 × 10^−10^–1 × 10^−7^	2.2 × 10^−11^	[118]
GO/ZnS	1 × 10^−10^–1 × 10^−7^	2.2 × 10^−11^	[119]
rGO-COO/g-C_3_N_4_	1 × 10^−8^–1 × 10^−3^	1.79 × 10^−9^	[120]
GQDs/AuNRs/PICA/F-Au	0.01–100 mg/mL	3.75 ng/mL^−1^	[121]

g-C_3_N_4_: graphitic-phase carbon nitride, PICA/F-Au: poly(indole-6-carboxylic acid)/flower-gold.
